# Epitope Profiling of Diphtheria Toxoid Provides Enhanced Monitoring for Consistency Testing during Manufacturing Process Changes

**DOI:** 10.3390/vaccines10050775

**Published:** 2022-05-13

**Authors:** Camille Houy, Marin Ming, Luciano Ettorre, Robbie Jin, Nemika Thangavadivel, Tricia Chen, Jin Su, Beata Gajewska

**Affiliations:** Department of Analytical Sciences, Sanofi Pasteur, Toronto, ON M2R 3T4, Canada; marin.ming@sanofi.com (M.M.); luciano.ettorre@sanofi.com (L.E.); robbie.jin@mail.utoronto.ca (R.J.); nemika.thangavadivel@sanofi.com (N.T.); tricia.chen@sanofi.com (T.C.); jin.su@sanofi.com (J.S.); beata.gajewska@sanofi.com (B.G.)

**Keywords:** quantitative epitope profiling, biolayer interferometry, diphtheria toxoid, vaccine, comparability, manufacturing process changes, antibody characterization

## Abstract

In the vaccine industry, multiple physicochemical, immunological, *in vitro* and *in vivo* analytical methods are applied throughout the manufacturing process to characterize and monitor the quality of vaccines. Presented here is the Single Epitope Antigenicity Test (SEAT), an innovative, quantitative epitope profiling method which provides an extended immunochemical analysis for diphtheria toxoid (DTxd) to be used for consistency testing during manufacturing process changes. The method uses BioLayer Interferometry (BLI) and a panel of monoclonal antibodies (mAbs) to independently assess nine individual antigenic sites of DTxd. The panel includes mAbs which are functional, bind distinct sites on DTxd and are able to distinguish intact DTxd from that which has been exposed to heat treatment. The SEAT method was qualified for precision, accuracy, and linearity, and was used to define a preliminary comparability range for DTxd made using the current manufacturing process. DTxd lots manufactured using alternate processes were assessed in the context of this range to determine the impact on DTxd antigenicity. Epitope profiling by SEAT provides quantitative information on the integrity of multiple important antigenic regions of DTxd, and therefore represents a valuable tool in a comprehensive analytical test package which can be used to support manufacturing process changes for vaccines.

## 1. Introduction

During vaccine development and for release of commercial products, a panel of physicochemical, immunological, *in vitro* and *in vivo* analytical methods is required at multiple stages of the manufacturing process [1]. These methods are used to characterize and monitor the safety, purity, potency, identity, and biological activity of the product [2,3,4]. Following development, the tests play an important role in ensuring batch to batch consistency for commercial products manufactured using a well-defined manufacturing process [5].

As a result of increased scientific understanding and improved technologies in a constantly evolving industry, vaccine manufacturers regularly update their manufacturing processes. This can be performed to increase the production scale, to comply with changes in regulatory requirements and/or to reduce the use of animal derived components. When such changes are made, it is critical to ensure that there is no adverse impact on the drug product. Hand in hand with updating production processes, it is incumbent upon manufacturers to develop and apply advanced analytical methods to assess their products. In the case of vaccine manufacturers, in addition to a panel of physicochemical methods used for most pharmaceutical products, it is important to apply immunochemical analyses to understand the impact of manufacturing changes on the immunological properties of the product [6].

Diphtheria toxin (DTxn) is manufactured by purification from cultured *Corynebacterium diphtheria* and detoxification with formaldehyde to generate Diphtheria toxoid (DTxd) before being adsorbed onto adjuvant for use in diphtheria vaccine products. As a process update for DTxn has recently been initiated for implementation in a new manufacturing facility, comparability studies must be conducted in order to assess the impact of the process modification on the DTxd. Typically, release tests for diphtheria vaccines include *in vivo* immunogenicity tests for antibody response and/or animal challenge models for protection. In addition, various immunochemical and physicochemical analyses at different stages of manufacturing can be applied in order to monitor the quality of the DTxd [7]. In the case of this process update, it was desirable to apply an extensive immunological analysis to monitor the effect of the process change on DTxd.

Antigenic sites, or epitopes, are the regions of a molecule which are recognized by antibodies in the adaptive immune response. For vaccines, it is critical to retain the antigenic sites across each manufacturing lot in order to retain the capacity of the vaccine to elicit a protective immune response. Manufacturing process changes have the potential to alter the integrity of DTxd epitopes, i.e., the primary structure for linear epitopes, or the secondary or tertiary structure in the case of conformational epitopes. Altered folding of the DTxd could also impact the accessibility of an epitope, by changing its degree of exposure on the surface of the molecule.

Current antigenicity tests use polyclonal antibodies (in the case of flocculation tests or polyclonal ELISA [8]). These provide important data for assessing overall antigenicity, as they report a single value which can be considered a congregate sum of all individual epitopes on the antigen. As a result, however, these assays could conceivably mask changes in single important epitope regions, i.e., the overall antigenicity may be only slightly affected even if there is a significant change in one particular region. The value of monitoring a collection of single epitopes during critical process changes is that it provides better resolution on individual regions of the molecule. In this study, we present the development of an innovative epitope profiling method which uses BioLayer Interferometry (BLI) and a panel of monoclonal antibodies (mAbs) to independently assess multiple antigenic sites of DTxd at the purified toxoid stage. Each mAb is utilized in a Single Epitope Antigenicity Test (SEAT) to monitor the integrity or accessibility of an individual epitope on DTxd. Together the panel of SEATs delivers an ‘epitope profile’ which provides comprehensive quantitative information on antigenic sites across the DTxd molecule. The SEAT is not intended to be used for the quantification of diphtheria toxoid itself, but rather as a means of monitoring individual epitopes compared to overall antigenic content.

BLI is a real time technology that uses the interference pattern of white light reflected from the surface of a biosensor where molecules are attached to assess kinetics of mAb-epitope binding [9]. Biosensor-based methods have been used to support a variety of vaccine research, development, and production studies including vaccine quality control [10], epitope design and epitope binning [11], characterization of pathogen mechanism of action and host immune response [12], antibody affinity [13] and therapeutics and clinical studies [14]. In this study, BLI was used for the final SEAT method, wherein immobilized mAbs on the biosensor are used to bind free, unlabeled DTxd in solution, as a means of assessing individual epitopes. BLI was also used as a screening and characterization tool for the selection of the mAbs used in the SEAT. Each candidate mAb was assessed for binding site (DTxn subunit binding sites and epitope binning) and functionality (capacity of the mAb to neutralize DTxn and/or to inhibit the binding of DTxn to its cellular target, heparin binding epidermal growth factor-like growth factor (HB-EGF)). In addition, the mAbs were assessed for their ability to detect changes in antigen that were induced by heat treatment. The SEAT assays were assessed for specificity, linearity, accuracy, and precision across a defined range of DTxd concentrations, and were used to assess DTxd manufactured using routine and alternative processes.

## 2. Materials and Methods

### 2.1. Material

#### 2.1.1. Antibodies

Murine mAbs were produced by Envigo (Indianapolis, IN, USA) using DTxd (Sanofi Pasteur, Toronto, ON, Canada) as antigen. Twelve DTxd-specific mAbs were selected using direct ELISA. Biotinylation of the mAbs was required for the immobilization of the mAbs on streptavidin biosensors. Biotinylation was performed at a molecular ratio of 1:3 mAb:biotin for 30 min using EZ-Link-NHS-PEG4 Biotin (Thermo 213229, Waltham, MA, USA), then excess free biotin was removed using Zeba Desalting spin columns, 7K MWCO (Thermo 89882).

#### 2.1.2. Diphtheria Toxin and Toxoid

Routine manufacturing lots of DTxn (purified from *Corynebacterium diphtheria*) and DTxd (formaldehyde-inactivated DTxn, with a purity of at least 1500 Lf/mg nitrogen) (Sanofi Pasteur) were used in the studies. DTxd generated using the routine process (process A) as well as those generated with an alternate purification process (process B), an alternate culture media and alternate purification process (process C), DTxd which was past expiry date (process A, 5 years post date of manufacture), and DTxd that had been exposed to heat (45 °C for up to 28 days or 60 °C for up to 14 days) were assessed. For the SEAT, a representative lot of DTxd (process A) was used as the reference standard. The concentration of DTxd used in this study is the Lf/mL content as determined by Ramon’s Flocculation method [15].

### 2.2. mAb Characterization Methods

#### 2.2.1. General BLI Method

Assay buffer (1X Kinetic Buffer-1X KB) consists of a 1/10 (*v*/*v*) dilution of 10X Kinetic Buffer (Sartorius 18-1105, Göttingen, Germany) in Phosphate Buffered Saline (PBS; 1X, pH 7.4; ThermoFisher 10010023). BLI assays were conducted at 30 °C with shake speed of 1000 rpm. Unless otherwise specified: at the start of each assay, biosensors were rehydrated for ≥10 min in assay buffer, and the first step of the assay was to establish an initial baseline in assay buffer. Anti-mouse IgG Fc Capture (AMC) biosensors (Sartorius 18-5088) were used to immobilize non-biotinylated mAbs, and streptavidin biosensors (Sartorius 18-5019) and high precision streptavidin (SAX) biosensors (Sartorius 18-5117) were used to immobilize biotinylated mAbs.

The signal from antigen association when an irrelevant mAb was bound to the probe was assessed during early experiments and was determined to be immeasurably low; therefore, subtraction of the non-specific binding signal was not applied for subsequent BLI experiments.

#### 2.2.2. Binning Experiment

The mAbs were tested in binning experiments using BLI. Briefly: a 300 s immobilization step of one biotinylated mAb (20 µg/mL) onto a streptavidin biosensor was followed by a 60 s wash step in 1X KB and then by a 600 s association step of DTxd (20 µg/mL in 1X KB). A second 60 s wash step in 1X KB was then performed to establish post-antigen baseline, followed by a 300 s association step of the second (non-biotinylated) mAb (20 µg/mL).

Results were aligned to the post-antigen baseline. If a binding signal of the second mAb to the DTxd was not observed, it was considered to be in the same bin as the first mAb. Results were confirmed with the position of first and second mAb reversed.

#### 2.2.3. Inhibition of mAb-DTxn Binding to HB-EGF

Binding of the mAbs to the HB-EGF binding site on DTxn was assessed using BLI: first, DTxn was pre-incubated with HB-EGF (Cedarlane, CLY100-180, Burlington, ON, Canada) for 60 min at 37 °C at a molecular ratio of 1:20 DTxn:HB-EGF. After a 600 s loading step of biotinylated mAbs (20 µg/mL) onto streptavidin biosensors, a 60 s wash step in 1X KB was performed to establish post-mAb baseline. Finally, a 600 s association step of the DTxn/HB-EGF complex or DTxn alone (15 µg/mL) was performed. Results were aligned to the post-mAb baseline. The biosensors were also exposed to HB-EGF alone to confirm the absence of non-specific binding of HB-EGF to the sensor.

#### 2.2.4. Toxin Neutralization Assay

All reagents were prepared in assay medium (DMEM plus GlutaMAX (Gibco 10564, Waltham, MA, USA), 1% penicillin/streptomycin (Gibco 15140-122), 1% gentamycin (Gibco 15710-072), 10% Fetal Bovine Serum (Hyclone SH3008403IR, Logan, UT, USA)) and all incubations were at 37 °C, 5% CO_2_, unless otherwise noted.

DTxn was prepared in twelve 2.5-fold serial dilutions starting at 2.375 × 10^−2^ Lf/mL and 75 µL of each dilution was transferred to the wells of a flat bottom 96-well tissue culture plate (Becton Dickinson, Franklin Lakes, NJ, USA). Then mAb (0.5 µg/125 µL/well) was added and plates were incubated for 1 h. Vero cells (ATCC, Manassas, VA, USA) were then added (1 × 10^4^ cells/50 µL/well) to wells with the toxin-mAb mixtures or to cell control wells with medium only (250 µL/well), and plates were incubated for four days. Medium was removed, neutral red (Sigma-Aldrich N2889 (St Louis, MO, USA), 0.5 mg/mL in DMEM plus GlutaMAX) was added (200 µL/well) and the plates were incubated for 3 h. Plates were washed once with D-PBS (Gibco 14190, 200 µL/well) prior to addition of 1% acetic acid (EMD Millipore AX0073-6, Burlington, VT, USA)/50% ethanol (Commercial Alcohols, Brampton, ON, Canada) (200 µL/well). Plates were incubated at RT for 20 min, shaken at 600 rpm for 1 min, and then absorbance (540 nm) was read on a spectrophotometer. The cell viability threshold was calculated as 50% of the average A_540_ of the cell control wells; wells with A_540_ above this value were considered positive for live cells. The neutralization capacity of the mAbs was correlated directly to the amount of toxin used rather than to an international antitoxin reference. The first well that was positive for live cells in the toxin dilution series corresponds to the neutralizing titre of the mAb: 1 U is the amount of antibody required to neutralize 1 Lf of toxin. For example, if the first live well in the toxin dilution series is the fifth well (4.56 × 10^−5^ Lf/well), this indicates that 4.56 × 10^−5^ Lf was neutralized by the 0.5 µg mAb present in that well; therefore, the neutralizing titre of that mAb is 4.56 × 10^−5^ U/5 × 10^−4^ mg = 9.12 × 10^−2^ U/mg.

#### 2.2.5. BLI Screening for mAb Sensitivity to Heat-Induced Degradation of DTxd

The ability of mAbs to distinguish native from altered epitopes on the surface of DTxd was assessed using BLI. Altered DTxd was generated by exposure to thermally accelerated degradation conditions of 60 °C for up to 14 days. Briefly: a 600 s association step of mAb (10 µg/mL) onto AMC biosensors was followed by a 150 s wash step in 1X KB to establish pre-DTxd baseline, and then a 700 s association step of DTxd or heat-treated DTxd (10 µg/mL) was performed. Results were aligned to the pre-DTxd baseline. The sensitivity of each mAb was determined by calculating the ratio of the binding rate for heat-treated DTxd to untreated DTxd. mAbs with increased sensitivity were considered to bind DTxd epitopes which are more susceptible to degradation by heat.

#### 2.2.6. Western Blot

The ability of mAbs to recognized DTxn subunit A or B and also their ability to bind heat-degraded DTxd was assessed using Western blot. Briefly, 1.5 µg of sample (untreated DTxn, untreated DTxd, DTxd treated 11 days at 60 °C and DTxd treated 1 day at 90 °C) was prepared in 1X LDS sample buffer (Invitrogen NP007, Waltham, MA, USA) and 1X reducing agent (Invitrogen NP009), incubated at 90 °C for 10 min, and electrophoresed with SeeBlue Plus2 pre-stained protein standard (Invitrogen LC5925) for 2 h 15 min at 100 V on a 10% Bis-Tris NuPAGE gel (Invitrogen NP0315) in 1X NuPAGE MOPS SDS running buffer (Invitrogen NP0001). The proteins were transferred to a PVDF membrane (Invitrogen IB401002) using iBlot^TM^ system (Invitrogen) at 20 V for 7 min. The membrane was blocked overnight at 4 °C in blocking solution (Western breeze kit, Invitrogen WB7103) and immunodetection with individual anti-DTxd mAbs (~0.15 µg/mL) was performed using the Western breeze kit.

For Coomassie staining, gels were run as described for Western blot experiments and the gels were developed using InstantBlue^®^ (Sigma-Aldrich ISB1L).

#### 2.2.7. Single Epitope Antigenicity Testing (SEAT)

##### SEAT Assay Design

Each SEAT was performed with one mAb: a 120 s loading step of biotinylated mAb (20 µg/mL) onto a SAX biosensor was followed by a 60 s wash step in 1X KB before a final 120 s association step of either DTxd test sample (in a 2-fold, 8-step serial dilution starting at 420 Lf/mL) or an in-house reference standard of DTxd (in a 2-fold, 7-step serial dilution starting at 42 Lf/mL plus one well containing 1X KB only, representing the “0” concentration of antigen).

The antigen association step was analyzed using the quantitation module of the Octet data analysis software (version 11, Sartorius). Octet readout was based on the initial slope of the antibody-analyte binding response curve at 120 s, with the low concentration and the zero concentration thresholds set at 0.0001. The initial slope data was then transferred to SoftMax Pro (SMP) software (version 5.4.1, Molecular Devices, San Jose, CA, USA) for quantitative analysis. SMP was chosen for analysis rather than Octet software as it provides enhanced curve fitting and is compliant for use in GMP/GLP labs; also, with the use of automated templates, SMP offers reduced time and error risk at the analysis stage.

4-PL curves with weighting of 1/y^2^ were fit to test samples and in-house reference standard, and DTxd was quantitated (Lf/mL) relative to the reference standard. Lack of non-parallelism was confirmed by slope ratio of sample to reference standard falling between 0.80 and 1.25. If necessary, aberrant values were masked to reduce overall %CV to ≤20%, with minimum of five points remaining after masking. Antigenicity of the sample was expressed in %Recovery as (observed/theoretical concentration*100) where the theoretical concentration is the Lf/mL content of the test sample as determined by Ramon’s Flocculation method [15]. Thus, the assay reports the concentration of an individual epitope relative to the overall antigen concentration.

##### SEAT Qualification

A total of nine SEATs, each using a single anti-DTxd mAb, were assessed at four DTxd concentration levels (1X, 0.5X, 0.25X and 0.1X, representing a starting concentration of 420, 210, 105, and 42 Lf/mL, respectively). DTxd sample prepared at each level was tested three times in independent assays, performed on different days, each assay with a different lot of biotinylated mAb and with a different plate layout, by a total of two analysts.

For linearity assessment, the observed values (in Lf/mL, based on SEAT measurement) and the theoretical values (concentration in Lf/mL measured by flocculation) were log10 transformed and fitted with a linear regression model. The goodness of fit was measured by the R-squared (R^2^) value. The slope of the linear regression model was also examined as part of the linearity assessment. Accuracy was evaluated by calculating %Recovery (observed DTxd/theoretical DTxd (Lf/mL)*100) at the 1X, 0.25X, 0.5X and 0.1X levels. Intermediate precision was calculated at the 1X, 0.25X, 0.5X and 0.1X levels by %CV of DTxd antigenicity (Lf/mL or %Recovery) obtained by testing the same samples a minimum of three times over three independent assays.

##### Determination of the Comparability Range

Each of the SEATs was used to assess nine lots of DTxd manufactured using the routine process (process A). The samples were assessed over multiple days, with multiple biotinylation batches for each mAb. The mean ± 3 standard deviations (SD) was calculated to define the ‘comparability range’ for each SEAT; any test sample for which the antigenicity falls within this range was considered comparable to material manufactured using the routine process. The SEATs were then used to analyze DTxd which had been manufactured using an alternate DTxn purification process and/or alternate culture media, as well as routine material which had passed expiry date.

## 3. Results and Discussion

### 3.1. mAb Characterization

#### 3.1.1. Determination of Binning Groups and Subunit Binding Sites

DTxn is a 58 kDa protein comprised of two subunits linked by disulfide bridges; the A subunit (21 kDa) mediates enzymatic activity, and the B subunit (37 kDa) binds to the cell receptor and mediates translocation of the A chain to the cytosol [16]. The DTxn subunit binding sites of twelve anti-DTxd mAbs were determined by Western blot (Table 1, Figure S1). Formaldehyde inactivation of DTxn into DTxd creates intramolecular cross-links between the A and B subunits, resulting in a single band visible at 58 kDa on an SDS-PAGE gel for DTxd, as opposed to three bands observed for DTxn (unreduced DTxd at 58 kDa and subunits A and B at 21 kDa and 58 kDa, respectively) (Figure S1). For DTxd, the broadened 58 kDa band is expected, likely due to a heterogeneous population of product formed by different formaldehyde induced intramolecular cross-links [17].

An epitope binning assessment was performed to further categorize the mAbs into four distinct groups or ‘bins’. The BLI sensorgram for a representative binning experiment and summary results for binning of all twelve mAbs are shown in Table S1 and Figure S2. All mAbs within a bin are considered to bind the same molecular region of DTxd, i.e., they either bind the exact same epitope or epitopes that are in very close proximity on the surface of the molecule. The mAbs which are in the same bin were not considered redundant if other characterization tests revealed that they have different properties. Together, the collection of mAbs span two antigenic regions in the A subunit and two in the B subunit, as summarized in Table 1.

#### 3.1.2. Assessment of mAb-DTxn Binding Inhibition by HB-EGF

**Figure 1:**
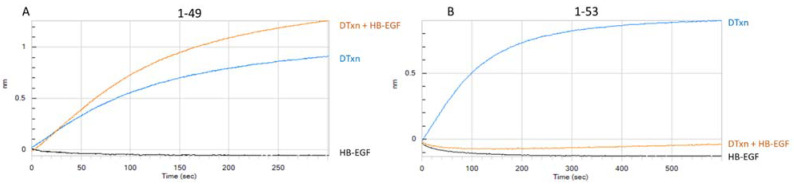
Inhibition of mAb binding to DTxd by HB-EGF. BLI signal upon association of DTxn to mAb 1-49 (**A**) or mAb 1-53 (**B**) immobilized onto the biosensor. Curves depict the association of: DTxn alone (blue); DTxn that has been pre-incubated with HB-EGF (60 min, 37 °C) (orange); HB-EGF alone (black).

HB-EGF is expressed on various human tissues and is a receptor for DTxn. DTxn binding to HB-EGF triggers receptor-mediated endocytosis of the toxin and is the first step of the mode of action of DTxn that eventually leads to the inactivation of protein synthesis and cell death by apoptosis [16,18]. mAbs that bind to the HB-EGF binding site of DTxn are often highly neutralizing due to their potential to prevent DTxn binding and entry to the target cell [19,20]. Therefore, maintaining the integrity of epitopes at the HB-EGF binding site on the DTxd is considered important in vaccine manufacturing, in order for the vaccine to elicit such neutralizing antibodies. In Figure 1, BLI profiles for two representative mAbs are shown. When DTxn was preincubated with HB-EGF, mAb 1-49 (loaded on the biosensor) was able to bind DTxn (Figure 1A), but mAb 1-53 was not (Figure 1B). This suggested that mAb 1-53 binds at or near the HB-EGF binding site of DTxn while the epitope for mAb 1-49 is located elsewhere on the DTxn molecule. The binding signal of mAb 1-49 to the DTxn-HB-EGF complex was slightly higher than to DTxn alone; this may be due to the increased size of the molecular complex including HB-EGF, or be reflective of a conformational change in DTxn upon binding to HB-EGF, both of which can affect the BLI signal. Results for all mAbs in the HB-EGF experiment, shown in Table 1, indicated that mAbs in binning region 2 are binding to, or near, the HB-EGF binding site of DTxn.

#### 3.1.3. Determination of Toxin Neutralization Capacity of the mAb Panel

The anti-DTxd mAbs were tested for their ability to neutralize DTxn in an in-house Vero cell DTxn neutralization assay (see Table 2). The mAbs were ranked based on their neutralizing capacity, with the strongest neutralizers being mAbs 2-18, 1-49 and 3-17. Moderate neutralizing capacity was observed for mAbs 1-53, 2-2, 2-25, and 3-45 and the remaining antibodies had weak neutralizing capacity. The neutralization capacity of the mAbs was tested in this study in order to rank them relative to each other and identify the strongest neutralizing mAbs. Of note, the strongest neutralizers were mAbs that did not inhibit DTxn binding to HB-EGF; furthermore, each of the strongest neutralizing mAbs bound to a different region of DTxd as indicated by binning region (see Table 1). This observation underlines the importance of monitoring multiple sites across DTxd for epitope integrity, as there are several important targets for neutralizing antibodies other than the receptor binding site [21].

#### 3.1.4. Determination of mAb Ability to Detect Thermally Induced Change in DTxd

**Figure 2:**
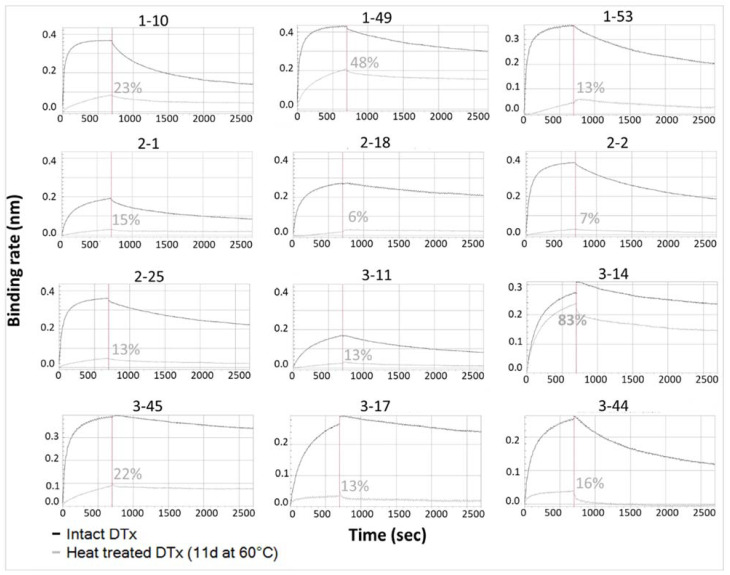
Binding curves of 12 individual mAbs to intact or degraded DTxd. Binding of each of 12 mAbs to untreated DTxd (black) or heat treated DTxd (11 d, 60 °C) (grey) is depicted. Association and dissociation were recorded in real time in nm. The binding rate is the height of the curve (nm) at the end of the association step (vertical line, 700 s). Binding rate of mAb to heat treated DTxd/untreated DTxd is shown (%).

Figure 2 depicts the binding of intact or heat treated DTxd to twelve anti-DTxd mAbs. Most of the mAbs demonstrated differential binding to intact vs heat treated DTxd, as observed with a partial or total signal reduction when DTxd had been heat treated for 11 days at 60 °C, compared to signal when binding to intact DTxd. This provided quantitative confirmation of the data obtained using Western blot (see Figure S1 for representative blots). Notably, mAb 3-14 was shown to have relatively poor sensitivity to heat degradation of DTxd, with binding signal only slightly reduced for heat treated compared to intact DTxd. Upon exposure to heat, unfolding of the toxoid can occur, and this has the potential to affect both the integrity of conformational epitopes as well as the accessibility of individual epitopes on the molecular surface [10,22]. The 60 °C heat treatment represents a thermally accelerated degradation condition that is known to cause a reduction in DTxd vaccine potency in animal protection models ([23] and confirmed in house (data not shown)). It was desirable to include heat sensitive epitopes in the epitope profile in order to detect these types of changes to molecular integrity that are known to affect vaccine potency.

### 3.2. Single Epitope Antigenicity Test (SEAT)

#### 3.2.1. Assay Development

Various conditions for a quantitative BLI assay were assessed, including the use of different biosensors, buffers, timing of assay steps, and dilution range for the mAb and the samples (data not shown). Different approaches were considered for data analysis, including the use of initial slope vs R equilibrium as the BLI readout, and the use of Octet vs SMP software for curve analysis and quantitation (data not shown). Ultimately a uniform method was defined for all mAbs, as the least variable and most sensitive method for antigenic quantitation. The method uses the initial slope of the mAb/DTxd association curve as readout, a 7-point dilution scheme for the reference standard and an 8-point dilution scheme for samples, a 4-PL curve fit for initial slope vs DTxd concentration, and slope ratio of sample/reference standard for assessment of parallelism (slope ratio between 0.80 and 1.25 was considered lack of evidence for non-parallelism).

#### 3.2.2. Assay Performance

Nine SEATs were qualified for use in DTxd epitope profiling. Three mAbs (mAb 2-1, 3-11 and 3-44) were excluded due to inconsistent performance in BLI during development (data not shown). Precision, accuracy, and linearity were assessed over a 10-fold range of DTxd concentrations (420 to 42 Lf/mL, representing 1X, 0.5X, 0.25X, and 0.1X the targeted starting concentration). The assays exhibited good intermediate precision (%CV <16% overall, <9% at the routine sample concentration of 1X), accuracy (recovery 72% to 115% overall, 91% to 115% at routine sample concentration), and linearity (R^2^ = 1.00 with slope close to 1). Table 3 summarizes the SEAT performance for the nine mAbs assessed.

#### 3.2.3. Sample Comparability Assessment

**Figure 3:**
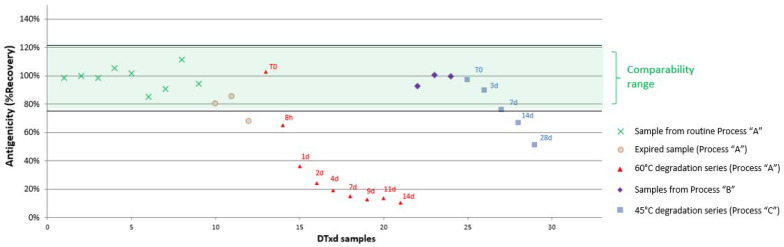
Antigenicity of intact, heat treated and expired DTxd samples in the SEAT using mAb 3-17. SEAT using mAb 3-17 was used to determine the antigenicity (%Recovery) of DTxd manufactured using the routine process (process A). Comparability range (green) is the average antigenicity (in %Recovery) ± 3 SD for nine lots of non-expired process A material (DTxd samples 1 to 9). Additional DTxd lots, including three expired process A lots (DTxd samples 10 to 12) and nine samples of a heat-treated process A lot (DTxd samples 13 to 21), as well as DTxd lots created using alternate manufacturing conditions (three lots, process B (samples 22 to 24) and five samples of a heat-treated lot from process C (samples 25 to 29)) were also tested, and are presented here for comparison.

**Figure 4:**
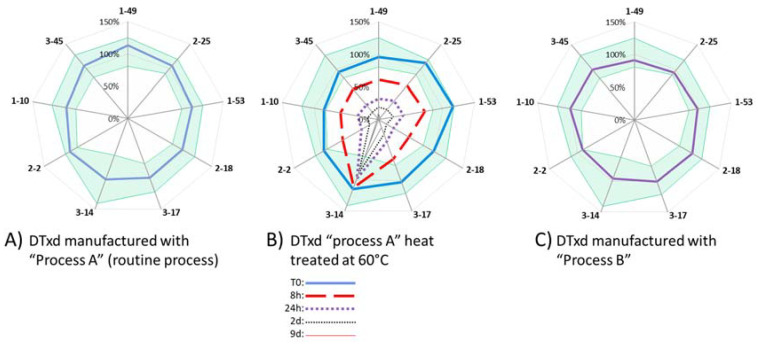
Epitope profile of DTxd material defined by SEAT using nine mAbs. SEAT using mAbs 1-49, 2-25, 1-53, 2-18, 3-17, 3-14, 2-2, 1-10, and 3-45 were used to determine the antigenicity (%Recovery) of DTxd. Comparability range (green area) was defined by the average antigenicity ± 3 SD for nine lots of DTxd manufactured using the routine process (process A). Epitope profiles are shown for (**A**) a representative lot of DTxd (‘process A’) (**B**) a single lot of process A material heat-treated at 60 °C for up to 9 d (**C**) DTxd manufactured using an alternate purification process (‘process B’).

For each SEAT, a comparability range was determined by the average antigenicity (expressed in %Recovery as “observed/theoretical concentration*100”) ± 3 SD for nine lots of DTxd manufactured using the routine process (process A). Any test sample for which the antigenicity falls within this range was considered comparable to material manufactured using the routine process. Figure 3 shows the results for sample comparability for a representative mAb 3-17 and confirms that the use of an alternate purification process (process B) or an alternate culture media and purification process (process C) did not have an impact on integrity or accessibility of the DTxd epitope bound by mAb 3-17. The analysis showed that DTxd which had been exposed to heat was no longer comparable to routine lots after 14 d at 45 °C or after 8 h at 60 °C, at which points decreased antigenicity was observed. It is unlikely that the heat treatment condition disturbed the covalent bonds of DTxd, since the cross-links formed on DTxd during the formaldehyde-induced detoxification are very stable and resistant to heat [24]. Therefore, any linear epitope present on the DTxd surface would likely remain intact. The decreased antigenicity observed with mAb 3-17 after heat treatment thus indicates that the epitope recognized by this antibody is likely conformational. Additionally, all of the lots of DTxd that had passed expiry date had lower antigenicity, and one of the lots was outside the comparability range assigned for mAb 3-17, demonstrating that this method could be used for detecting molecular changes on the DTxd surface over time. The complete epitope profile for DTxd, using all nine mAbs, is shown in Figure 4. The epitope profile of two different routine process lots (Figure 4A,B T0) and one alternate process lot (Figure 4C) are depicted. Each lot falls within the defined comparability range for each mAb; however, differences in the antigenicity for each epitope can be observed. The reduction in antigenicity when DTxd is exposed to heat is depicted in Figure 4B, confirming that the assay is able to detect heat-induced epitope changes on the DTxd molecule with all of the mAbs except mAb 3-14. mAb 3-14 also had a relatively wide comparability range for current process material (Table 3), indicating a high degree of variability at this epitope that is unrelated to process change; therefore, this mAb is not an ideal candidate for use in comparability testing. For the purpose of assay development and proof of concept, nine lots of DTxd were assessed to determine the comparability range for DTxd in this study. To apply the SEAT for assessment of process change, it is recommended to establish a product comparability range based on as many representative lots as required to obtain significant statistical power. For a higher throughput assay, mAbs within the same bin which consistently give the same information for all types of material tested may be eliminated.

## 4. Conclusions

A collection of anti-DTxd mAbs were characterized and used to develop a panel of SEATs which together can be used to define a comprehensive epitope profile of DTxd. Together, the mAbs are able to detect heat-induced molecular alteration of DTxd, they bind regions of DTxd which are functionally important in terms of DTxn neutralization, and they are located on different areas of the DTxd molecule. The SEAT method is quantitative and has been demonstrated to show accuracy (%Recovery between 72 and 115), precision (%CV ≤ 16) and linearity (R^2^ = 1.00) across the range of 420 to 42 Lf/mL, and represents a valuable tool for determining the impact of different process method changes on antigenic integrity and accessibility at multiple sites. Antigenicity is reported as the quantity of each specific epitope in relation to overall DTxd content. Efforts are ongoing in the development of a two-mAb sandwich ELISA for use in antigenicity testing of diphtheria vaccine products, with a goal to ultimately serve as a potency assay for product release [9]. The SEAT method presented here would complement such a high throughput ELISA in circumstances when a more detailed assessment of a product is desired. It provides greater resolution for individual epitopes than can be obtained using existing antigenicity tests (e.g., flocculation or polyclonal ELISA). By providing antigenicity information from multiple important sites across the target molecule, the SEAT profile can also play an important role in reducing the need for animal testing to monitor overall immunogenicity. The SEAT is well suited for application in the ‘consistency approach’ for vaccine testing, whereby thorough characterization of a vaccine using relevant analytical methods can be used to generate a product profile that allows the assessment of batch-to-batch quality [5].

## Figures and Tables

**Table 1:** Characterization summary of 12 anti-DTxd monoclonal antibodies.

mAb	Clone ID	Binding Site on DTxn (Subunit A or B)	Bin	Distinguishes Native from Heat Stressed DTxd	Inhibits DTxn Binding to HB-EGF Target	Neutralization Capacity
1-10	1-10.3.1.5.10	B	2	Yes	Yes	weak
1-49	1-49.1.2.8.6.3.8	A	1	Yes	No	strong
1-53	1-53.3.2.4.6.6	B	2	Yes	Yes	moderate
2-1	2-1.3.21	B	2	Yes	inconsistent	weak
2-18	2-18.1.8.8.3	A	3	Yes	No	strong
2-2	2-2.2.11	A	3	Yes	No	moderate
2-25	2-25.1.21.9.7.4.7	B	2	Yes	Yes	moderate
3-11	3-11.1.9	B	4	Yes	No	weak
3-14	3-14.1.1	B	4	No	inconsistent	weak
3-17	3-17.1.2	B	4	Yes	No	strong
3-44	3-44.2.7	A	3	Yes	No	weak
3-45	3-45.2.3	A	3	Yes	No	moderate

**Table 2:** Neutralizing capacities of anti-DTxd mAbs. Anti-DTxd mAbs were tested in a Vero cell diphtheria toxin neutralization assay to determine their neutralizing titres (endpoint assay, one unit of antibody neutralizes one Lf of toxin). Assay was repeated up to five times for each mAb (limited due to reagent availability). Results were consistent for all repeats.

mAb	Neutralizing Titre (×10^−3^ U/mg)	Category	*n*
1-10	<1	weak	2
1-49	10–100	strong	4
1-53	1–9	moderate	2
2-1	<1	weak	2
2-18	10–100	strong	5
2-2	1–9	moderate	1
2-25	1–9	moderate	4
3-11	<1	weak	1
3-14	<1	weak	1
3-17	10–100	strong	1
3-44	<1	weak	1
3-45	1–9	moderate	1

**Table 3:** Summary of the SEAT performance assessment.

Quality Check	Sample/ Parameter	1-49	1-53	2-25	2-18	3-14	3-17	1-10	2-2	3-45
**Intermediate Precision** (%CV, *n* = 3)	1X	7%	8%	2%	4%	4%	4%	8%	9%	7%
	0.5X	4%	3%	3%	14%	6%	1%	7%	5%	3%
	0.25X	9%	2%	2%	10%	10%	6%	5%	2%	3%
	0.1X	4%	4%	6%	9%	16%	9%	2% ^1^	9%	4%
**Accuracy** (%Recovery range, *n* = 3)	1X	96–111%	94–108%	99–104%	98–105%	99–108%	98–107%	91–105%	97–113%	101–115%
	0.5X	100–108%	95–101%	97–102%	80–105%	92–102%	96–97%	93–106%	98–107%	98–105%
	0.25X	93–110%	93–97%	93–96%	84–103%	86–104%	90–101%	93–103%	96–99%	97–103%
	0.1X	95–103%	87–93%	86–96%	80–95%	72–100%	81–96%	90–92% ^1^	87–103%	92–99%
**Linearity** (1X, 0.5X, 0.25X, 0.1X)	R^2^	1.00	1.00	1.00	1.00	1.00	1.00	1.00	1.00	1.00
	Slope	1.02	1.06	1.05	1.05	1.07	1.06	1.02	1.03	1.04
**Sensitive to Heat Induced Degradation ^2^**	N/Ap	Yes	Yes	Yes	Yes	poor ^3^	Yes	Yes	Yes	Yes
**Comparability Range** (%Recovery)	N/Ap	[80–125%]	[85–118%]	[90–119%]	[80–119%]	[61–141%]	[75–122%]	[82–120%]	[91–112%]	[83–128%]

^1^ Only two of three repetitions yielded reportable value due to failure of parallelism. These two values were used to estimate accuracy and precision. ^2^ Sensitivity defined as antigenicity for DTxd treated at 60 °C for 8 h being outside of the comparability range for the mAb (see Section 3.2.3). ^3^ Only the 14 d sample was outside the comparability range.

## Data Availability

The data presented in this study are available in this article or in the supplementary material. Raw data and some early development data are not included for the sake of brevity; however, it can be available on request from the corresponding author.

## Supplementary Materials

The following supporting information can be downloaded at: https://www.mdpi.com/article/10.3390/vaccines10050775/s1, Figure S1: Sensitivity to heat-induced degradation of DTxd and DTxn subunit binding determination for 3 anti-DTxd mAbs; Figure S2: Representative sensorgram with 9 different primary mAbs on the biosensor, tested in binning experiment with secondary mAb 3–44, Table S1: mAb binning experiments.
