# Supplementary material for: Epitope Profiling of Diphtheria Toxoid Provides Enhanced Monitoring for Consistency Testing during Manufacturing Process Changes
Source: Vaccines (Basel). 2022 May 13;10(5):775. doi: 10.3390/vaccines10050775 (PMC9147534; doi:10.3390/vaccines10050775)
Supplement: Supplementary file 1 [file vaccines-10-00775-s001.zip › vaccines-1675290-supplementary.pdf]

Supplementary Figures and Tables:

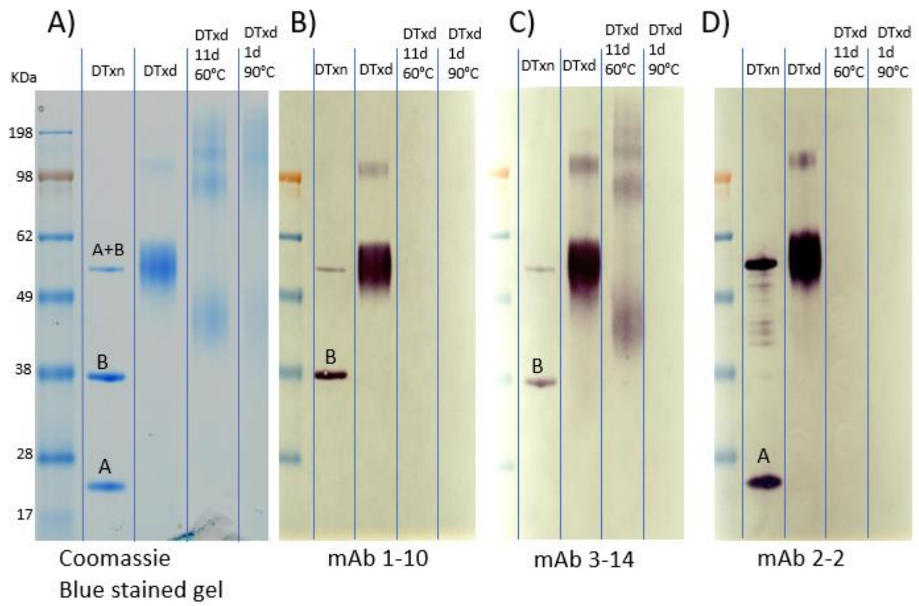

Figure S1: Sensitivity to heat-induced degradation of DTxd and DTxn subunit binding determination for 3 anti-DTxd mAbs

A)Total protein profile of DTxn, DTxd and heated treated DTxd (11 days at 60°C and 1 day at 90°C) is represented on the Coomassie stained SDS-PAGE gel (denaturing conditions). The corresponding Western blots performed with mAbs 1-10, 3-14 and 2-2 are represented respectively in **B**), **C**) and **D**), where mAb 1-10 and 3-14 are representative of mAbs binding to the B subunit of DTxn, and mAb 2-2 binds the A subunit. Neither mAb 1-10 nor 2-2 bind to DTxd which has been partially degraded by heat treatment, in contrast to mAb 3-14 which binds to both untreated DTxd and DTxd.

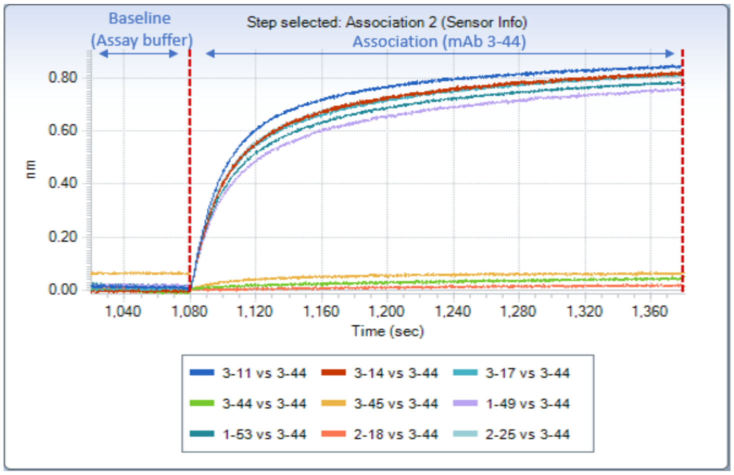

Figure S2: Representative sensorgram with 9 different primary mAbs on the biosensor, tested in binning experiment with secondary mAb 3-44

Primary mAbs 3-11, 3-14, 3-17, 3-44, 3-45, 1-49, 1-53, 2-18 and 2-25 were immobilized onto the biosensor, followed by association of DTxd (not pictured), and then a baseline was established in assay buffer followed by the association of the secondary mAb 3-44 (pictured above). Secondary mAb 3-44 does not bind to the DTxd when it is captured by mAbs 3-45, 2-18, or 3-44 (as indicated with the yellow, orange and green association curves), but binds when the DTxd is captured by the other mAbs (indicated by binding curves in the remaining colours). Any pair of mAbs which cannot simultaneously bind to DTxd are considered to be in the same bin.

Table S1: mAb binning experiments

Primary mAbs were immobilized onto the biosensor, followed by association of DTxd, and then association of the secondary mAbs. Green (+) indicates mAb pairs which can bind to DTxd simultaneously; red (-) indicates mAb pairs which cannot bind simultaneously; +/- indicates reduced binding for second mAb; yellow indicates pairs which exhibit binding inhibition in one orientation of the mAb pair but not the reverse. The binning determination was performed in two sets of experiments, as indicated by the two individual tables A) and B).

A) First Experiment

		mAb in solution (secondary)						
		1-49	1-10	1-53	2-1	2-25	2-18	2-2
immobilized mAb (primary)	1-49	- **Bin 1**	+	+	+	+	+	+
	1-10	+	-	-	-	-	+	+
	1-53	+	-	- **Bin 2**	-	-	+	+
	2-1	+	-	-	-	-	+	+
	2-25	+	-	-	-	-	+	+
	2-18	+	+	+	+	+	- **Bin 3**	-
	2-2	+	+	+	+	+	-	-

B) Second Experiment

		mAb in solution (secondary)								
		1-49	1-53	2-25	2-18	3-44	3-45	3-11	3-14	3-17
immobilized mAb (primary)	1-49	- **Bin 1**	+	+	+	+	+	+	+	+
	1-53	+	-	- **Bin 2**	+	+	+	+	+	+
	2-25	+	-	-	+	+	+	+	+	+
	2-18	+	+	+	-	-	-	+	+	+
	3-44	+	+	+	-	- **Bin 3**	+/-	+	+	+
	3-45	+	+	+	-	-	-	+	+	+
	3-11	+	+	+	+	+	+	-	+	+
	3-14	+	+	+	+	+	+	-	- **Bin 4**	-
	3-17	+	+	+	+	+	+	-	-	-
